# Fermentation Effect on Volatile Evolution of Plant-Based Dry-Cured Sausages

**DOI:** 10.3390/foods15020342

**Published:** 2026-01-17

**Authors:** José María Martín-Miguélez, Josué Delgado, Alberto González-Mohino, Lary Souza Olegario

**Affiliations:** 1 Higiene y Seguridad Alimentaria, Instituto de Investigación de Carne y Productos Cárnicos (IProCar), Facultad de Veterinaria, Universidad de Extremadura, Avda. de las Ciencias s/n, 10003 Cáceres, Spain; jmmm@unex.es; 2 Tecnología de los Alimentos, Instituto de Investigación de Carne y Productos Cárnicos (IProCar), Facultad de Veterinaria, Universidad de Extremadura, Avda. de las Ciencias s/n, 10003 Cáceres, Spain; albertogj@unex.es (A.G.-M.); laryolegario@unex.es (L.S.O.)

**Keywords:** aroma profile, lactic acid bacteria, *Lacticaseibacillus casei*, *Latilactobacillus sakei*, off-flavor mitigation

## Abstract

This study evaluates the effect of fermentation on the volatile composition of plant-based dry-cured sausages. The goal was to understand how different lactic acid bacteria (LAB) strains influence the aroma profile during ripening. Five experimental groups were tested, including uninoculated controls and sausages inoculated with selected LAB strains or a commercial starter. A total of 51 volatile compounds were identified and tracked over an 11-day fermentation period using HS-SPME-GC-MS. Results showed that LAB fermentation reduced compounds associated with off-flavors, such as aldehydes and sulfur compounds, and promoted the formation of volatiles responsible for pleasant aromas like buttery and fruity notes. Specific LAB strains, especially *Lacticaseibacillus casei* 116, showed strong potential in improving the volatile profile of plant-based meat analogs. These findings suggest that fermentation using selected LAB can enhance the sensory quality of plant-based sausages, helping them better mimic traditional meat products.

## 1. Introduction

Recent years have witnessed notable shifts in dietary patterns, particularly within developed nations and among individuals with higher household incomes, who are increasingly opting for organic products and are reducing their intake of meat as well as foods containing artificial additives [1,2,3]. This trend has contributed to the rise of plant-based analogs in the market, which try to sensorially mimic traditional meat-based products [4]. However, the processing to which plant-based raw materials are submitted to obtain a meat-like texture develops beany flavor in the final product [5]. Recent studies in plant-based matrices have shown that LAB fermentation can decrease lipid-derived aldehydes associated with beany and grassy notes and, at the same time, promote the formation of ketones, acids, and other volatiles typically linked to meat-like aromas [6]. In contrast, most of this work has focused on liquid or soft systems, and information on structured, dry-fermented plant-based systems remains scarce [7,8,9]. For plant-based dry-cured sausages, available research has mainly addressed safety, basic quality attributes, and overall acceptability, but a detailed characterization of how LAB fermentation modulates volatile profiles over ripening is still missing [10,11]. Nowadays, different technologies are currently being considered to avoid the off-flavor formation in meat analogs, though fermentation is the most promising due to its advantages in applicability and affordability [12]. Therefore, there is a need to clarify how defined LAB strains, compared with spontaneous or commercial fermentations, drive the time-course evolution of volatile compounds in plant-based dry-cured sausages and whether they can modulate the aroma profile towards a more meat-like character.

To obtain plant-based analogs more similar in flavor and texture to animal-derived products, lactic acid bacteria (LAB) are being used to remove off-flavors, eliminate antinutrients, and improve nutritional content [13,14]. The LAB metabolic activity on plant-based matrices is becoming widely studied for their reduction in aldehyde content, usually related to bean flavor, and the increase in ketones or other compounds traditionally found in meat products [15]. In plant-based dry-cured sausages, fermentation and the utilization of specific microorganisms, such as LAB, have been evaluated as acceptability enhancers, and previous work on this same analog has described its physicochemical, microbial, and sensory characteristics during ripening [10]. However, those studies did not include a systematic characterization of the volatile profile and only provided indirect information on aroma through sensory assessment. On the other hand, the positive impact of LAB has been widely studied in meat-based sausages for their ability to enhance safety, texture, and flavor through metabolic activities that generate volatile compounds [16,17]. LAB are one of the main microbial groups found in dry-cured sausages due to the environmental and physicochemical characteristics of these kinds of products [18]. Thus, the utilization of LAB to develop the aromatic profile of plant-based sausages might become an achievable strategy to increase consumer acceptance [19]. The present work implements different inoculation schemes (uninoculated control, single-strain inoculations, co-inoculation, and a commercial starter) by monitoring in detail the time-course evolution of volatile compounds throughout ripening, thereby directly linking LAB fermentation strategies to aroma development in a plant-based dry-cured sausage.

Volatile composition plays a crucial role in determining the sensory quality of foods, as it directly contributes to their aroma and flavor [20]. Consequently, it is usually related to consumer flavor liking [21]. The volatile profile is often considered an indicator of product quality and can even relate plant-based analogs to meat flavor [22]. Therefore, monitoring volatile composition is not only essential for understanding the sensory attributes of plant-based analogs but can also serve as a valuable parameter to assess flavor quality and consumer liking.

The participation of LAB in the formation of a meat-like aromatic profile in plant-based products is not yet fully understood. In particular, there is limited information on strain-specific effects in plant-based matrices, on how different LAB species modulate distinct families of volatile compounds over time, and on whether these changes can consistently shift the profile towards meat-like notes rather than merely suppressing plant-related off-flavors. However, it is well known that different LAB strains might influence the final aromatic profile of plant-based analogs [12]. The present study will help expand the yet limited research on this topic. It was hypothesized that selected LAB strains would differentially drive the evolution of volatile compounds in a plant-based dry-cured sausage and that specific strains, particularly *Lacticaseibacillus casei* 116 and *Latilactobacillus sakei* 205, would reduce off-flavor markers while promoting volatiles associated with meat-like, buttery, and fruity notes. The main objective is to compare the effect of different LAB strains on the volatile compound evolution of plant-based dry-cured sausages through ripening time. The findings will improve the understanding of whether LAB fermentation can produce a volatile composition more similar to that of meat products than spontaneous fermentation. Furthermore, it can guide the creation of products that better meet consumer expectations for flavor and aroma.

## 2. Materials and Methods

### 2.1. Microbial Cultures

A commercial *Latilactobacillus curvatus* strain (Amerex, Madrid, Spain), utilized by the manufacturer of the meat analog assessed in this study, was employed as part of the starter culture in combination with catalase-positive Gram-positive cocci (*Staphylococcus carnosus*). Additional LAB strains were sourced from the culture collection maintained by the Food Hygiene and Safety research group at the University of Extremadura (*Lacticaseibacillus casei* 116 and *Latilactobacillus sakei* 205). These isolates originated from “Torta del Casar” cheese and “salchichón”, a traditional dry-fermented sausage, respectively [23]. They have been selected due to their previously demonstrated inhibitory activity against *Listeria monocytogenes* in the matrix evaluated in the present research [10].

All cultures were revived from frozen stocks stored at −80 °C in their respective media supplemented with 20% (*w*/*v*) glycerol (Thermo Fisher Scientific, Waltham, MA, USA). For inoculum preparation, LAB strains were incubated in Man, Rogosa, and Sharpe (MRS) broth (Conda Pronadisa, Madrid, Spain) at 30 °C for 48 h, respectively. Following incubation, cultures were centrifuged at 10,621× *g* for 5 min in 50 mL polypropylene tubes (Scharlau Chemie S.A., Barcelona, Spain). The supernatant was discarded, and the cell pellet was resuspended in phosphate-buffered saline (PBS; prepared with 0.32 g sodium dihydrogen phosphate (Scharlau Chemie S.A., Barcelona, Spain), 1.09 g disodium hydrogen phosphate (Scharlau Chemie S.A., Barcelona, Spain), and 9 g NaCl (Labbox Labware S.L., Barcelona, Spain) per liter of distilled water). Initial cell concentrations of LAB were determined on day 0 of each experiment by plating on MRS agar for LAB, for each experimental group evaluated in a previously published research [10]. The batter was inoculated to reach targeted levels of approximately 8 log CFU/g at stuffing for each experimental group. Thus, each LAB-inoculated group was adjusted to target 8 log CFU/g of the inoculated strains: *L. casei* 116, *L. sakei* 205, and co-inoculation (*L. casei* 116 + *L. sakei* 205; 7.7 log CFU/g per strain, total 8 log CFU/g), while the commercial starter followed manufacturer specifications (~8 log CFU/g LAB). The uninoculated control group received no deliberate inoculation.

### 2.2. Plant-Based Dry-Cured Sausages Elaboration

For this study, a ready-to-eat (RTE) plant-based dry-cured sausage was utilized as the model product. Production was carried out at the University of Extremadura, following the formulation supplied by the company responsible for commercializing the meat analog under investigation. The sausage was prepared using a blend of potato, olive oil, shea butter, pea protein, dried red pepper, garlic, paprika, salt, vegetable fibers, and a plant-derived casing (United Caro, Granada, Spain). No additional curing agents (nitrite/nitrate), sugars, or starter nutrients were included beyond these listed ingredients, ensuring a clean-label formulation that relies on intrinsic plant carbohydrates for LAB fermentation. Physicochemical, microbial, and sensory analyses of this analog have been previously reported [10,11].

Five experimental groups were evaluated to understand the aromatic impact of different microorganisms on the fermented sausage. For each group, three sausages were prepared from the same batter preparation. A control group without any starter culture (experimental group 1), *L. casei* 116 inoculation (experimental group 2), *L. sakei* 205 inoculation (experimental group 3), *L. casei* 116 and *L. sakei* 205 co-inoculation (experimental group 4), and a commercial starter culture (experimental group 5). They were individually hung and ripened in a drying chamber AN501SC (Infrico, Lucena, Spain) at 9.0 °C ± 1.0 and 80.0% ± 2.0 relative humidity for 11 days with samplings on day 0, 2, 5, 8, and 11. At each sampling point, volatile analysis was performed on individual sausages from each biological triplicate (n = 3), to capture batch-to-batch variability.

### 2.3. Volatile Identification

The analysis of volatile compounds in plant-based dry-cured sausages was performed in triplicate for each experimental group at five different time points: days 0, 2, 5, 8, and 11. Volatile extraction was carried out using solid-phase microextraction (SPME). For each sample, 3.5 g was placed in 20 mL glass vials. The samples were equilibrated by heating at 40 °C for 25 min prior to extraction. Volatiles were then adsorbed onto a 50/30 µm Divinylbenzene/Carboxen/Polydimethylsiloxane (DVB/CAR/PDMS) fiber (Merck, Darmstadt, Germany).

Following extraction, the analytes were desorbed and analyzed using gas chromatography coupled with mass spectrometry (GC-MS). The instrumentation consisted of an Agilent 6890 GC system (Santa Clara, CA, USA) equipped with a DB1 capillary column (60 m × 0.25 mm × 1.2 µm), interfaced to an Agilent 5975C mass selective detector. The GC oven temperature was initially set at 40 °C for 5 min, then ramped at 5 °C/min to 230 °C, and subsequently increased at 25 °C/min to a final temperature of 250 °C. Desorption of volatiles from the fiber was performed at 250 °C for 15 min (splitless mode), with the transfer line maintained at 280 °C. Helium (Air Liquide, Madrid, Spain) was used as the carrier gas at a constant flow rate of 1.2 mL/min. Mass spectra were obtained by electronic impact at 70 eV, collecting data at a rate of 4,5 scan s^−1^ over the *m*/*z* range 35–350.

Mass spectrometric detection was conducted in full scan mode, covering a mass range of 50–350 amu. Chromatographic peaks were manually integrated by a single analyst using consistent criteria: baseline selection from valley-to-valley between peaks, mass spectral similarity threshold >80%, peak area > 1000, and deconvolution treatment of overlapping peaks. Co-eluting peaks were resolved by dropping perpendicular to the baseline. Compounds were identified when mass spectral quality was >80% against the NIST/EPA/NIH library (National Institute of Standards and Technology, Gaithersburg, MD, USA) and linear retention indices agreed within ±10 units of literature values for the utilized column. All samples were processed by the same analyst to ensure comparability and minimize inter-operator variability. Additionally, linear retention indices were calculated and compared to those obtained from a standard mixture of C6–C20 n-alkanes (Sigma Aldrich Co., St. Louis, MO, USA) analyzed under identical chromatographic conditions to confirm compound identities. This strategy has been evaluated as a strong method to verify the molecule identity [24,25]. Results are reported as relative peak areas (AU; arbitrary units) and cannot be interpreted as absolute concentrations.

### 2.4. Statistical Analysis

For each volatile compound, a two-way ANOVA was performed with fermentation TIME (5 levels: days 0, 2, 5, 8, and 11) and inoculation TREATMENT (5 levels: control, *L. casei* 116, *L. sakei* 205, co-inoculation, and commercial starter) as fixed factors, including the TIME × TREATMENT interaction term. This statistical analysis was performed using IBM SPSS Statistics v.22 software (IBM Co., New York, NY, USA). Further, the statistical analysis of the collected data was performed using Tukey’s honest significant difference (HSD) test in GraphPad Prism version 10.1.1 (San Diego, CA, USA). Statistical significance was established at a *p*-value threshold of 0.05 or lower. MetaboAnalyst 6.0 (https://www.metaboanalyst.ca/) (Montreal, QC, Canada) was utilized to perform the K means clustering and the heatmap classification using the 51 individual volatile compounds as variables (not families). Peak area data (AU × 10^6^) were auto-scaled prior to analysis. The dataset consisted of 25 observations (5 experimental groups × 5 timepoints, n = 3 biological replicates each) and 51 volatile compound variables, demonstrating similarities and differences in volatile composition among the plant-based dry-cured sausages.

## 3. Results and Discussion

The 51 volatile compounds identified in the samples analyzed were separated into families: acids (8 compounds), alcohols (2 compounds), aldehydes (9 compounds), ketones (10 compounds), hydrocarbons (2 compounds), esters (4 compounds), terpenes (2 compounds), sulfur compounds (3 compounds), and other compounds (11 compounds). They have been displayed in Supplementary Table S1.

**Figure 1:**
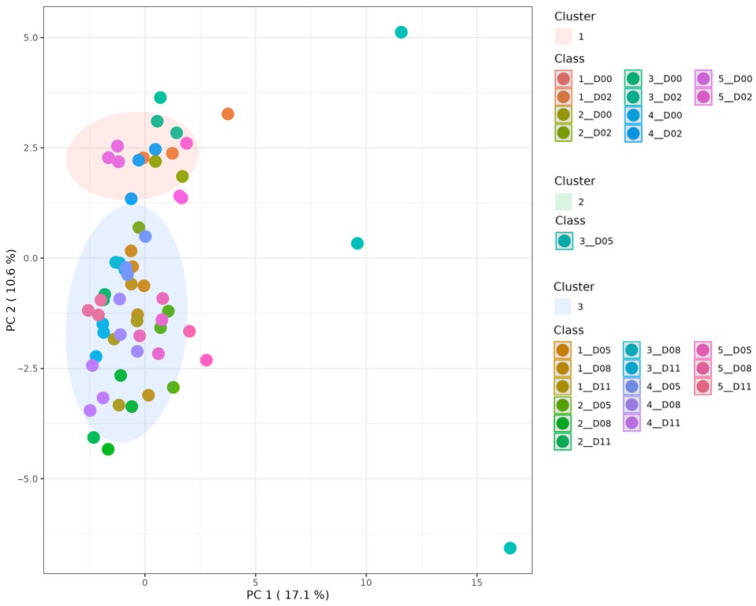
Principal Component Analysis (PCA) displaying 3 clusters for the plant-based dry-cured sausages analyzed. Uninoculated control (1), *Lacticaseibacillus casei* 116 (2), *Latilactobacillus sakei* 205 (3), *L. casei* 116 + *L. sakei* 205 (4), and commercial starter culture (5). Days 0 (D00), 2 (D02), 5 (D05), 8 (D08), and 11 (D11).

Firstly, a K means clustering was performed to understand whether fermentation showed any effect on the dry-cured sausages evaluated. Figure 1 shows a clear differentiation between the samples on initial and final ripening days. Cluster 1 was associated with the samples of every experimental group on days 0 and 2 of ripening. Cluster 2 was only formed by the samples of the experimental group inoculated with *L. sakei* 205 on day 5. Cluster 3 contained samples of the experimental groups and the ripening days left. Thus, fermentation can generate differences in the plant-based sausages evaluated, though the process may reduce initial compounds or generate new ones in early or late stages of fermentation.

### 3.1. Volatile Compounds Reduced During Fermentation

The initial identification of several compounds originally from the raw materials employed has been eliminated through the fermentation process, in general, or the activity of specific LAB strains. Different acids, aldehydes, ketones, sulfur, and other compounds responsible for off-flavors have been removed from the plant-based dry-cured sausages.

**Figure 2:**
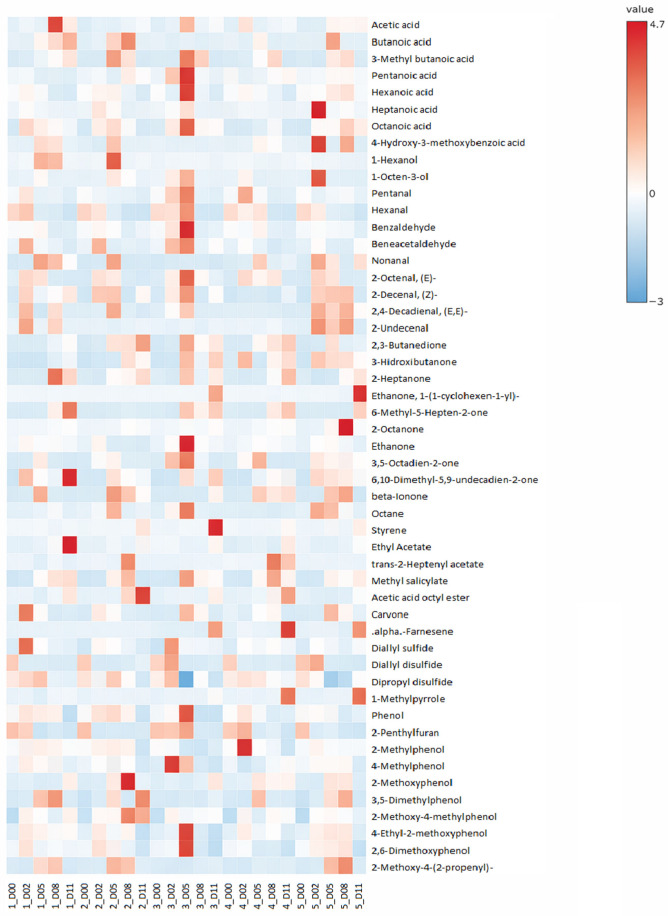
Heat map displaying the volatile compounds for the plant-based dry-cured sausages analyzed. Peak area data (AU × 10^6^) were autoscaled prior to visualization, with red indicating values >0 (above row mean) and blue <0 (below row mean). Uninoculated control (1), *Lacticaseibacillus casei* 116 (2), *Latilactobacillus sakei* 205 (3), *L. casei* 116 + *L. sakei* 205 (4), and commercial starter culture (5). Days 0 (D00), 2 (D02), 5 (D05), 8 (D08), and 11 (D11).

Hexanoic acid was found to decrease during the fermentation period from 0.26 to 0.03–0.20 AU × 10^6^ (*p* ≤ 0.05). The experimental group inoculated with the commercial LAB displayed the highest concentration since day 5 of sampling (*p* ≤ 0.05), as can be observed in Figure 2. This starter might produce hexanoic acid as a result of its metabolism. This fact may contribute to a lower product acceptability, as hexanoic acid is usually related to a fatty off-note in leguminous grains, one of the ingredients of the sausage evaluated [26].

Pentanal and hexanal have been found in the initial days of fermentation with concentrations of 0.05 and 0.65 AU × 10^6^, respectively. However, they have not been detected on days 8 and 11, respectively. Ripening time reduces their concentration in any experimental group sampled (*p* ≤ 0.05). Specifically, in the case of hexanal, the batches inoculated with LAB have displayed lower concentrations of this aldehyde (*p* ≤ 0.05). These aldehydes have been identified as the main off-flavor drivers in legumes, usually identified with grass and beany odor [27,28]. This finding agrees with the ones found by other authors who observed the reduction of off-flavor aldehydes through LAB fermentation of plant raw materials [15]. Thus, the reduction of these aldehydes during fermentation is an interesting aspect since it may lead to improved product acceptability.

The concentration reduction in other aldehydes, such as benzaldehyde, through the fermentation time, is also worth mentioning (*p* ≤ 0.05). Its initial concentration was 0.09 AU × 10^6^. However, on the last sampling day, it was around 0.02 AU × 10^6^. No differences were found between experimental groups, though it can be observed that fermentation processing allowed the reduction of these compounds. While aldehydes as benzaldehyde is associated with pleasant almond-like notes, and benzeneacetaldehyde contributes honey/floral character, both compounds have been identified as off-flavor contributors in plant-based protein matrices due to their association with bitter/oxidized notes [29,30]. Fermentation can become an interesting process to reduce the off-flavors in plant-based sausages, as was evaluated.

Moreover, the action of some specific LAB strains, such as *L. casei* 116, can be observed in the concentration evolution of compounds such as 2-pyrrolyl methyl ketone. This ketone was initially found at a concentration of 0.07 AU × 10^6^, and its concentration decreased under the detection levels from day 5 when *L. casei* 116 was present.

The sulfur compounds identified in the present research were found in the highest concentrations on the initial day (*p* ≤ 0.05), as can be observed in Figure 2. While sulfur volatiles like diallyl sulfide and disulfides can contribute desirable garlic/onion-like aroma notes at trace levels in meat products, their presence in plant-based matrices is frequently associated with off-flavor and can be related to plant-based raw materials [26]. These compounds can be related to plant-based raw materials, like garlic, and their reduction during fermentation is usually found in meat sausages too [31]. Diallyl sulfide, diallyl disulfide, and dipropyl disulfide displayed original concentrations of 0.09, 0.48, and 0.07 AU × 10^6^. No differences were found between the sampled experimental groups, though the fermentation process reduced the diallyl sulfide and dipropyl disulfide concentrations up to 0.06 and 0.05 AU × 10^6^, respectively (*p* ≤ 0.05). Diallyl disulfide was reduced to non-detectable concentrations since day 5 of sampling. Diallyl sulfide and diallyl disulfide are compounds originally found in ingredients such as fresh garlic, utilized in the present experimental analog, but tend to disappear through short-term fermentation [32]. Sulfur compounds are usually related to egg, onion, and cabbage-like odors, so the reported reduction within the fermentation process in the present study might be considered as a positive aspect to avoid off-flavors [15].

The fermentation effect over the initial volatile profile has been displayed in compounds such as phenol and 2-penthylfuran, which had initial concentrations of 0.13 and 2.23 AU × 10^6^, respectively, and decreased until they were not detected on the final day of fermentation in any of the sampled experimental groups (*p* ≤ 0.05). Phenol contributes medicinal/phenolic off-notes characteristic of unprocessed legumes, while 2-penthylfuran is a specific marker of lipid oxidation in soy/pea proteins [33]. These compounds are related to the raw plant protein flavor present in pea protein isolates, and their substantial reduction during fermentation likely mitigates these legume-specific off-flavors, contributing to an aroma profile more suitable for plant-based analogs. On the other hand, there have been other compounds whose concentration has also decreased throughout fermentation time (*p* ≤ 0.05), though the metabolic activity of LAB has favored the non-detection of certain compounds. Two-methylphenol, 4-methylphenol, 4-ethyl-2-methoxyphenol, and 2,6-dimethoxyphenol were not detected on the final day of fermentation in the experimental group inoculated with *L. casei* 116. They can be related to smoky, phenolic, or spicy notes, and other authors have also highlighted how these compounds can be eliminated from different raw materials through microbial fermentation [34,35,36]. Microbial fermentation might improve the final product flavor by reducing the off-flavor of plant-based protein.

The spontaneous fermentation process, driven by wild contaminating LAB that reached ~9.6 log CFU/g by day 6 in the uninoculated control group [10], also demonstrated its capacity to reduce concentrations of volatile compounds associated with undesirable sensory attributes in plant-based raw materials. However, contributions from other microbial groups and endogenous plant enzymes likely also participated in these transformations. The inoculation of specific LAB, such as *L. casei* 116, amplified the degradation of specific volatiles like ethenone and phenolic compounds, reinforcing the relevance of starter culture selection in the development of plant-based dry-cured meat analogs.

### 3.2. Volatile Compounds in Early Fermentation

The production of volatile compounds in the early fermentation days is mainly driven by acids such as heptanoic or octanoic acid. These compounds were not detected on the initial day and then appeared on days 2 and 5 in all experimental groups, reaching concentrations of 0.13 ± 0.11 AU × 10^6^ for heptanoic acid and 0.10 ± 0.02 AU × 10^6^ for octanoic acid, with no significant differences between treatments at each timepoint. These acids are related to cheese and rancid fat odors, being usually correlated to a pH decrease during fermentation [33,37]. With no observed differences between the experimental groups, the origin might be related to the microbial population of the evaluated sausages. Other acids, such as butanoic and 3-methyl butanoic acid, have also been found in the middle sampling days, resulting from the hydrolysis of lipids usually related to pulse spoilage [38]. However, in the present study, it has been observed that the inoculation of LAB has prevented the continuous increase in these volatile compounds through fermentation time, as it was observed in the control experimental group (*p* ≤ 0.05).

The alcohols identified in the fermented sausages were not found in the initial or the final samples. 1-Hexanol and 1-octen-3-ol were developed during fermentation, reaching concentrations of 0.35 ± 0.07 AU × 10^6^ and 0.48 ± 0.71 AU × 10^6^, respectively. 1-Hexanol likely originates from the enzymatic reduction of hexanal by endogenous legume alcohol dehydrogenases present in the initial batter, while 1-octen-3-ol results from both lipoxygenase activity and microbial metabolism contamination microorganisms [27,39]. These alcohols are commonly associated with green/fungal notes and sometimes considered deterioration markers in stored products, though their transient appearance during active fermentation represents normal metabolic intermediates rather than quality compromise, as concentrations declined by day 11 across all treatments [38,39].

Aldehydes, such as nonanal, 2-octenal, 2-decenal, 2,4-decadienal, and 2-undecenal, were also only detected during the middle sampling days, without a significant increase or decrease in their concentration, but with the lack of compound detection on the initial day. Their concentration reached values as 0.25, 0.07, 0.12, 0.07, and 0.04 AU × 10^6^, respectively. These aldehydes contribute to grassy, beany, or fatty odors considered off-flavors, and are usually formed through fatty acids’ enzymatic degradation [26,40].

During the early stages of fermentation, the production of acids and alcohols and the disappearance of initial off-flavor aldehydes/ketones reflected ongoing microbial and enzymatic activity. However, a sensory assessment would be required, as volatile evolution alone does not guarantee consumer overall liking.

### 3.3. Volatile Compounds in Late Fermentation

The key volatile compounds responsible for fermentation flavor are those whose concentration increased during the sampling time. In this late fermentation group, some vinegar-like compounds, such as acetic acid, are responsible for acid flavor and pungency [40], and have increased their concentration during fermentation time in every experimental group sampled from 0.39 up to 0.94 AU × 10^6^ (*p* ≤ 0.05). Moreover, the experimental group inoculated with the commercial LAB displayed the highest acetic acid concentration on the final fermentation day (*p* ≤ 0.05). While acetic acid contributes desirable fermented/vinegar notes that enhance savory complexity in dry-cured products at moderate levels, excessive concentrations can impart overly sharp, vinegary off-notes that may reduce overall sensory balance [41]. This trade-off highlights the importance of strain selection to achieve optimal acetic acid production without sensory dominance [42].

The selection of LAB is also highlighted in the case of ketones, such as 6-methyl-5-hepten-2-one, whose concentration increased during the fermentation time in any experimental group but the one inoculated with *L. casei* 116 (*p* ≤ 0.05). This methylated ketone originates from free fatty acid breakdown, and the metabolic activity of *L. casei* 116 might affect its formation [40]. 6-methyl-5-hepten-2-one contributes citrus/lemon-like notes at trace levels but can impart rancid/soapy off-notes at elevated concentrations typical of plant-based matrices [43]. However, *L. casei* 116 did not affect the formation of off-flavor ketones like 2-heptanone or 2-octanone, increasing with the fermentation time in every experimental group (*p* ≤ 0.05), up to a concentration of 0.35 AU × 10^6^ on the final day, and is related to soapy flavor [30]. On the other hand, other ketones such as 2,3-butanedione or 3-hydroxybutanone are responsible for the buttery flavor [14], and fermentation increased the concentration of these compounds up to 0.24 and 0.66 AU × 10^6^ on the final sampling day (*p* ≤ 0.05). Specifically, 2,3-butanedione displayed higher values on the final fermentation day in the experimental groups in which *L. casei* 116 was inoculated (*p* ≤ 0.05). This treatment displayed up to 12 times higher concentration than the 2,3-butanedione in the inoculated culture compared to the commercial culture.

In the present study, ester formation did not appear to be strongly strain dependent. Esters have been detected in the late days of ripening in any of the evaluated experimental groups. Ethyl acetate, trans-2-heptenyl acetate, methyl salicylate, and acetic acid octyl ester were not detected in the initial days of fermentation, but after day 5. This fact might be associated with the reduction of other volatile compounds into esters during the ripening time, as it has been investigated by other authors [44,45]. The lipid oxidation during the fermentation period might be behind the ester’s formation, and the inoculation of LAB does not seem to have a meaningful effect [46]. However, these compounds have a great impact on meat products with fruity and floral flavors [47]. Alpha-farnesene was a terpene only found in the experimental groups inoculated and co-inoculated with *L. sakei* 205, and the commercial one on day 11 of ripening. This compound likely originates from the biotransformation of spices of plant-based protein by *L. sakei* terpene cyclase activity, as alpha-farnesene is not a major constituent of the raw ingredients but emerges during late-stage microbial metabolism, though it is usually found in flowers and fruits [48]. It is related to a kiwifruit-like aroma [49,50].

The present study demonstrates that fermentation significantly modifies the volatile composition of plant-based dry-cured sausages, reducing concentrations of aldehydes, ketones, sulfur, and phenolic compounds associated with beany/grassy off-notes while promoting the formation of esters, buttery ketones, and terpenes. These compositional shifts are consistent with literature-reported odor descriptors, suggesting improved aroma potential. However, actual sensory quality and consumer acceptability remain unverified in this study, representing a key limitation that requires dedicated sensory analysis for confirmation. Future studies need to be addressed to understand the acceptability of the new flavor profile in different plant-based analogs, but this possibility could participate in the clean-label strategy already pursued by industries and researchers.

## 4. Conclusions

Lactic acid bacteria fermentation significantly modified the volatile profile of plant-based dry-cured sausages over 11 days of ripening. Across all LAB treatments, fermentation reduced aldehydes, sulfur compounds, and phenolic off-note contributors, while promoting esters and acids. *L. casei* 116 uniquely suppressed 6-methyl-5-hepten-2-one accumulation while maximizing buttery ketones (2,3-butanedione +14-fold vs. other treatments; *p* ≤ 0.05). These results indicate that fermentation with selected LAB strains can steer the volatile profile toward compounds typically associated with more favorable aromas (buttery, fruity notes) rather than plant-derived off-notes. However, confirmatory sensory testing is required to validate actual consumer perception. This approach holds prospective potential to support the development of more appealing plant-based meat alternatives without added artificial flavorings, as demonstrated by this clean-label formulation, thereby contributing to clean-label strategies.

## Data Availability

The original contributions presented in this study are included in the article/Supplementary Materials. Further inquiries can be directed to the corresponding author.

## Supplementary Materials

The following supporting information can be downloaded at: https://www.mdpi.com/article/10.3390/foods15020342/s1, Table S1: Peak area count ± SD (×10^6^) of volatile compounds identified in plant-based dry-cured sausages samples by HS-SPME/GC-MS.

## Abbreviations

The following abbreviations are used in this manuscript:

LAB	Lactic acid bacteria
