# Supplementary material for: Fermentation Effect on Volatile Evolution of Plant-Based Dry-Cured Sausages
Source: Foods. 2026 Jan 17;15(2):342. doi: 10.3390/foods15020342 (PMC12840526; doi:10.3390/foods15020342)
Supplement: Supplementary file 1 [file foods-15-00342-s001.zip › foods-4046626-supplementary.pdf]

Supplementary Table S1. Peak area count ± SD (× 10^6) of volatile compounds identified in plant-based dry-cured sausages samples by HS-SPME/GC-MS.

Day	Id.	0					2					5					8					11					P_A		
Group		1	2	3	4	5	1	2	3	4	5	1	2	3	4	5	1	2	3	4	5	1	2	3	4	5	TIME	TREATMENT	TIME × TREATMENT
Acids																													
Acetic acid	MS	0.39 ± 0.42	0.39 ± 0.42	0.39 ± 0.42	0.39 ± 0.42	0.39 ± 0.42	0.22 ± 0.04	0.21 ± 0.02	0.24 ± 0.00	1.14 ± 1.67	0.15 ± 0.01	0.14 ± 0.02	0.20 ± 0.00	0.28 ± 0.02	0.16 ± 0.02	0.67 ± 0.15	0.54 ± 0.25	0.28 ± 0.01	0.31 ± 0.05	0.43 ± 0.05	0.68 ± 0.01	0.94 ± 0.50	0.36 ± 0.03	0.47 ± 0.04	0.34 ± 0.06	0.68 ± 0.04	0.013	0.000	0.032
Butanoic acid	MS	n.d.	n.d.	n.d.	n.d.	n.d.	n.d.	n.d.	n.d.	n.d.	n.d.	0.02 ± 0.00^{a}	0.03 ± 0.01^{a,b}	n.d.	0.02 ± 0.00^{a}	0.05 ± 0.00^{c}	0.03 ± 0.00^{b}	0.06 ± 0.01^{c}	n.d.	n.d.	n.d.	0.04 ± 0.01^{b}	n.d.	n.d.	n.d.	n.d.	0.768	0.001	0.000
3-Methyl butanoic acid	MS	n.d.	n.d.	n.d.	n.d.	n.d.	n.d.	n.d.	n.d.	n.d.	n.d.	0.02 ± 0.00^{a}	0.02 ± 0.00^{a,b}	0.02 ± 0.01^{a,b}	0.02 ± 0.00^{a,b}	0.04 ± 0.00^{b,c}	0.02 ± 0.01^{a,b}	0.04 ± 0.00^{a,b,c}	0.05 ± 0.02^{c}	0.04 ± 0.01^{c}	0.04 ± 0.01^{b,c}	0.04 ± 0.02^{a,b,c}	n.d.	n.d.	n.d.	n.d.	0.044	0.000	0.096
Pentanoic acid	MS	0.03 ± 0.00^{a,b}	0.03 ± 0.00^{a,b}	0.03 ± 0.00^{a,b}	0.03 ± 0.00^{a,b}	0.03 ± 0.00^{a,b}	0.02 ± 0.01^{a}	0.04 ± 0.03^{a,b,c}	0.13 ± 0.03^{e}	n.d.	0.03 ± 0.00^{a}	n.d.	n.d.	0.08 ± 0.02^{d}	0.02 ± 0.00^{a}	0.06 ± 0.01^{b,c,d}	n.d.	0.02 ± 0.01^{a}	0.03 ± 0.00^{a,b}	0.04 ± 0.00^{a,b,c}	0.06 ± 0.01^{c,d}	n.d.	0.04 ± 0.01^{a,b,c}	0.02 ± 0.00^{a}	0.01 ± 0.00^{a}	0.03 ± 0.01^{a}	0.018	0.000	0.033
Hexanoic acid	MS	0.26 ± 0.04^{e,f,g}	0.26 ± 0.04^{e,f,g}	0.26 ± 0.04^{e,f,g}	0.26 ± 0.04^{e,f,g}	0.26 ± 0.04^{e,f,g}	0.35 ± 0.03^{g}	0.34 ± 0.03^{g}	0.35 ± 0.01^{g}	0.34 ± 0.06^{g}	0.33 ± 0.03^{g}	0.30 ± 0.02^{f,g}	0.34 ± 0.02^{g}	0.26 ± 0.02^{e,f,g}	0.25 ± 0.03^{e,f,g}	0.60 ± 0.05^{i}	0.06 ± 0.02^{a,b}	0.10 ± 0.02^{a,b,c,d}	0.18 ± 0.04^{c,d,e,f}	0.17 ± 0.05^{b,c,d,e}	0.48 ± 0.08^{h}	0.03 ± 0.01^{a}	n.d.	0.08 ± 0.02^{a,b,c}	0.03 ± 0.01^{a}	0.20 ± 0.06^{d,e,f}	0.000	0.000	0.044

Heptanoic acid	MS, KI	n.d.	n.d.	n.d.	n.d.	n.d.	0.03 ± 0.02 ^{a,b}	0.10 ± 0.13 ^{a,b}	0.02 ± 0.00 ^{a}	0.02 ± 0.00 ^{a}	0.13 ± 0.11 ^{b}	0.01 ± 0.00	0.02 ± 0.01 ^{a}	0.01 ± 0.00 ^{a}	n.d.	0.02 ± 0.01 ^{a}	n.d.	n.d.	n.d.	n.d.	0.02 ± 0.01 ^{a}	n.d.	n.d.	n.d.	n.d.	n.d.	0.001	0.000	0.005
Octanoic acid	MS	n.d.	n.d.	n.d.	n.d.	n.d.	0.09 ± 0.03 ^{b}	0.07 ± 0.01 ^{a,b}	0.07 ± 0.02 ^{a,b}	n.d.	0.07 ± 0.01 ^{a,b}	0.06 ± 0.01 ^{a,b}	0.10 ± 0.02 ^{b}	0.06 ± 0.01 ^{a,b}	0.03 ± 0.00 ^{a}	0.05 ± 0.01 ^{a,b}	0.04 ± 0.03 ^{a}	0.04 ± 0.04 ^{a}	0.05 ± 0.00 ^{a,b}	0.04 ± 0.01 ^{a}	0.09 ± 0.03 ^{b}	0.04 ± 0.01 ^{a}	n.d.	0.04 ± 0.01 ^{a}	n.d.	0.06 ± 0.01 ^{a,b}	0.274	0.326	0.023
4-Hydroxy-3-methoxybenzoic acid	MS	n.d.	n.d.	n.d.	n.d.	n.d.	n.d.	n.d.	n.d.	n.d.	0.22 ± 0.16 ^{b}	0.05 ± 0.03 ^{a}	0.08 ± 0.03 ^{a}	n.d.	0.03 ± 0.00 ^{a}	n.d.	0.05 ± 0.02 ^{a}	n.d.	n.d.	0.03 ± 0.00 ^{a}	0.13 ± 0.11 ^{a,b}	n.d.	n.d.	n.d.	n.d.	n.d.	0.501	0.674	0.011
Alcohols																													
1-Hexanol	MS, KI	n.d.	n.d.	n.d.	n.d.	n.d.	n.d.	n.d.	n.d.	n.d.	n.d.	0.25 ± 0.03 ^{b}	0.35 ± 0.07 ^{c}	n.d.	n.d.	n.d.	0.15 ± 0.07 ^{a}	n.d.	n.d.	n.d.	n.d.	n.d.	n.d.	n.d.	n.d.	n.d.	0.000	0.000	0.000
1-Octen-3-ol	MS, KI	n.d.	n.d.	n.d.	n.d.	n.d.	n.d.	n.d.	0.09 ± 0.01	0.08 ± 0.01	0.48 ± 0.71	0.10 ± 0.01 ^{b}	0.09 ± 0.01 ^{b}	0.04 ± 0.00 ^{a}	n.d.	n.d.	n.d.	0.05 ± 0.01	n.d.	n.d.	n.d.	n.d.	n.d.	n.d.	n.d.	n.d.	0.000	0.000	0.000
Aldehydes																													
Pentanal	MS, KI	0.05 ± 0.00 ^{a}	0.05 ± 0.00 ^{a}	0.05 ± 0.00 ^{a}	0.05 ± 0.00 ^{a}	0.05 ± 0.00	0.17 ± 0.22	0.03 ± 0.02	0.28 ± 0.07	0.13 ± 0.12	n.d.	n.d.	n.d.	0.14 ± 0.03	0.02 ± 0.00	n.d.	n.d.	n.d.	n.d.	n.d.	n.d.	n.d.	n.d.	n.d.	n.d.	n.d.	-	-	-
Hexanal	MS, KI	0.65 ± 0.01 ^{i}	0.65 ± 0.01 ^{i}	0.65 ± 0.01 ^{i}	0.65 ± 0.01 ^{i}	0.65 ± 0.01 ^{i}	0.56 ± 0.03 ^{h,i}	0.55 ± 0.01 ^{h,i}	0.44 ± 0.05 ^{e,f}	0.45 ± 0.11 ^{e,f,g}	0.51 ± 0.01 ^{f,g,h}	0.30 ± 0.06 ^{c,d}	0.11 ± 0.05 ^{b}	0.22 ± 0.03 ^{c}	0.38 ± 0.04 ^{d,e}	0.10 ± 0.04 ^{a,b}	0.02 ± 0.00 ^{a}	n.d.	n.d.	n.d.	0.06 ± 0.01 ^{a,b}	n.d.	n.d.	n.d.	n.d.	n.d.	0.000	0.001	0.000
Benzaldehyde	MS	0.09 ± 0.01 ^{b,c}	0.09 ± 0.01 ^{b,c}	0.09 ± 0.01 ^{b,c}	0.09 ± 0.01 ^{b,c}	0.09 ± 0.01 ^{b,c}	0.18 ± 0.01 ^{e}	0.19 ± 0.02 ^{e}	0.17 ± 0.04 ^{e}	0.19 ± 0.02 ^{e}	0.16 ± 0.01 ^{d,e}	0.15 ± 0.01 ^{d,e}	0.09 ± 0.04 ^{b,c}	0.10 ± 0.02 ^{c}	0.16 ± 0.01 ^{d,e}	0.11 ± 0.02 ^{c,d}	n.d.	n.d.	0.02 ± 0.00 ^{a}	n.d.	0.04 ± 0.02 ^{a,b}	n.d.	0.02 ± 0.01 ^{a}	0.02 ± 0.00 ^{a}	n.d.	0.02 ± 0.00 ^{a}	0.000	0.018	0.005

Benzeneacetaldehyde	MS	0.04 ± 0.02	0.04 ± 0.02	0.04 ± 0.02	0.04 ± 0.02	0.04 ± 0.02	0.35 ± 0.45	0.37 ± 0.52	0.32 ± 0.42	0.07 ± 0.05	0.40 ± 0.55	0.05 ± 0.03	n.d.	0.17 ± 0.21	0.05 ± 0.01	0.03 ± 0.01	0.03 ± 0.01	n.d.	0.02 ± 0.00	n.d.	0.02 ± 0.00	n.d.	n.d.	0.02 ± 0.00	n.d.	0.02 ± 0.00	0.259	0.000	0.469
Nonanal	MS	n.d.	n.d.	n.d.	n.d.	n.d.	n.d.	n.d.	n.d.	n.d.	0.25 ± 0.02 ^{c}	0.25 ± 0.01 ^{c}	0.24 ± 0.02 ^{c}	n.d.	0.23 ± 0.00 ^{c}	0.25 ± 0.01 ^{c}	0.18 ± 0.01 ^{b}	n.d.	n.d.	n.d.	n.d.	0.06 ± 0.00 ^{a}	n.d.	n.d.	n.d.	0.15 ± 0.02 ^{b}	0.000	0.000	0.000
2-Octenal, (E)-	MS, KI	n.d.	n.d.	n.d.	n.d.	n.d.	0.07 ± 0.01 ^{c}	0.07 ± 0.02 ^{c}	0.07 ± 0.00 ^{c}	0.07 ± 0.00 ^{c}	0.07 ± 0.01 ^{c}	0.05 ± 0.00 ^{b,c}	0.05 ± 0.01 ^{b,c}	0.05 ± 0.01 ^{b,c}	0.05 ± 0.00 ^{b,c}	0.06 ± 0.01 ^{b,c}	n.d.	n.d.	0.04 ± 0.02 ^{a,b}	0.01 ± 0.00 ^{a}	n.d.	n.d.	n.d.	n.d.	n.d.	n.d.	0.000	0.665	0.665
2-Decenal, (Z)-	MS	n.d.	n.d.	n.d.	n.d.	n.d.	0.10 ± 0.06	0.11 ± 0.08	0.07 ± 0.02	n.d.	0.10 ± 0.06	0.07 ± 0.05	0.12 ± 0.06	0.03 ± 0.01	n.d.	0.11 ± 0.06	0.04 ± 0.02	n.d.	n.d.	n.d.	0.12 ± 0.07	0.07 ± 0.06	0.06 ± 0.02	0.03 ± 0.00	0.02 ± 0.01	n.d.	0.062	0.117	0.144
2,4-Decadienal, (E,E)-	MS, KI	n.d.	n.d.	n.d.	n.d.	n.d.	0.07 ± 0.06	n.d.	0.03 ± 0.01	n.d.	0.07 ± 0.05	n.d.	0.07 ± 0.04	0.01 ± 0.01	n.d.	0.07 ± 0.04	0.04 ± 0.00	n.d.	n.d.	n.d.	0.06 ± 0.04	n.d.	n.d.	n.d.	n.d.	0.01 ± 0.00	0.447	0.089	0.037
2-Undecenal	MS, KI	n.d.	n.d.	n.d.	n.d.	n.d.	0.04 ± 0.03	n.d.	n.d.	n.d.	0.04 ± 0.01	n.d.	n.d.	n.d.	n.d.	0.03 ± 0.02	0.02 ± 0.00	n.d.	n.d.	n.d.	0.04 ± 0.03	n.d.	n.d.	n.d.	n.d.	n.d.	0.159	0.001	0.244
Ketones																													
2,3-Butanedione	MS	n.d.	n.d.	n.d.	n.d.	n.d.	n.d.	n.d.	n.d.	n.d.	n.d.	n.d.	0.09 ± 0.01 ^{a,b,c}	0.03 ± 0.00 ^{a,b}	0.04 ± 0.00 ^{a,b}	0.11 ± 0.01 ^{b,c}	0.02 ± 0.00 ^{a}	0.11 ± 0.03 ^{b,c}	0.07 ± 0.01 ^{a,b,c}	0.10 ± 0.01 ^{a,b,c}	0.02 ± 0.00 ^{a}	0.12 ± 0.13 ^{b,c}	0.24 ± 0.02 ^{d}	0.08 ± 0.00 ^{a,b,c}	0.15 ± 0.01 ^{c}	0.02 ± 0.00 ^{a}	0.025	0.000	0.000
3-Hidroxibutanone	MS, KI	n.d.	n.d.	n.d.	n.d.	n.d.	n.d.	n.d.	n.d.	0.25 ± 0.07 ^{a,b,c,d}	0.17 ± 0.03 ^{a,b,c}	n.d.	n.d.	0.16 ± 0.11 ^{a,b,c}	0.04 ± 0.01 ^{a}	0.72 ± 0.27 ^{f}	0.07 ± 0.04 ^{a,b}	0.51 ± 0.24 ^{c,d,e,f}	0.32 ± 0.22 ^{a,b,c,d,e}	0.55 ± 0.13 ^{d,e,f}	0.41 ± 0.12 ^{b,c,d,e,f}	0.20 ± 0.19 ^{a,b,c,d}	0.35 ± 0.07 ^{a,b,c,d,e}	0.66 ± 0.15 ^{e,f}	0.62 ± 0.12 ^{e,f}	0.24 ± 0.06 ^{a,b,c,d}	0.238	0.000	0.002
2-Heptanone	MS, KI	0.04 ± 0.00 ^{a}	0.04 ± 0.00 ^{a}	0.04 ± 0.00 ^{a}	0.04 ± 0.00 ^{a}	0.04 ± 0.00 ^{a}	0.03 ± 0.00 ^{a}	0.03 ± 0.00 ^{a}	0.03 ± 0.00 ^{a}	0.03 ± 0.00 ^{a}	0.03 ± 0.00 ^{a}	0.03 ± 0.00 ^{a}	0.04 ± 0.01 ^{a}	0.05 ± 0.00 ^{a,b}	0.03 ± 0.00 ^{a}	0.03 ± 0.00 ^{a}	0.14 ± 0.02 ^{a,b,c,d}	0.16 ± 0.03 ^{b,c,d,e}	0.08 ± 0.00 ^{a,b,c}	0.11 ± 0.05 ^{a,b,c,d}	0.11 ± 0.06 ^{a,b,c,d}	0.35 ± 0.01 ^{f}	0.27 ± 0.04 ^{e,f}	0.19 ± 0.04 ^{c,d,e}	0.35 ± 0.15 ^{f}	0.21 ± 0.02 ^{d,e}	0.000	0.288	0.420
Ethanone, 1-(1-cyclohexen-1-yl)-	MS	n.d.	n.d.	n.d.	n.d.	n.d.	n.d.	n.d.	n.d.	n.d.	n.d.	n.d.	n.d.	n.d.	n.d.	n.d.	n.d.	n.d.	n.d.	n.d.	n.d.	n.d.	n.d.	0.03 ± 0.00	n.d.	0.03 ± 0.00	-	-	-
6-Methyl-5-Hepten-2-one	MS, KI	n.d.	n.d.	n.d.	n.d.	n.d.	n.d.	n.d.	n.d.	n.d.	n.d.	n.d.	n.d.	0.02 ± 0.01 ^{a}	n.d.	n.d.	0.03 ± 0.00 ^{a,b}	n.d.	0.04 ± 0.01 ^{a,b,c}	0.04 ± 0.00 ^{a,b,c}	n.d.	0.10 ± 0.04 ^{d}	n.d.	0.05 ± 0.01 ^{b,c}	0.06 ± 0.00 ^{c}	0.03 ± 0.01 ^{a,b}	0.000	0.000	0.000

2-Octanone	MS	n.d.	n.d.	n.d.	n.d.	n.d.	n.d.	n.d.	n.d.	n.d.	n.d.	n.d.	n.d.	0.01 ± 0.00 ^{a}	0.03 ± 0.01 ^{b,c}	0.02 ± 0.00 ^{a,b}	0.03 ± 0.01 ^{b,c}	0.05 ± 0.01 ^{d}	0.02 ± 0.00 ^{a,b}	0.03 ± 0.01 ^{b,c}	0.02 ± 0.00 ^{a,b}	0.04 ± 0.02 ^{c,d}	0.03 ± 0.00 ^{b,c}	0.02 ± 0.00 ^{a,b,c}	0.03 ± 0.00 ^{b,c}	0.02 ± 0.01 ^{a,b}	0.000	0.078	0.000
Ethanone	MS	0.07 ± 0.01 ^{a,b,c,d}	0.07 ± 0.01 ^{a,b,c,d}	0.07 ± 0.01 ^{a,b,c,d}	0.07 ± 0.01 ^{a,b,c,d}	0.07 ± 0.01 ^{a,b,c,d}	0.10 ± 0.02 ^{d}	0.10 ± 0.02 ^{c,d}	0.09 ± 0.00 ^{b,c,d}	0.09 ± 0.01 ^{b,c,d}	0.09 ± 0.01 ^{b,c,d}	0.09 ± 0.01 ^{b,c,d}	n.d.	0.08 ± 0.00 ^{a,b,c,d}	0.08 ± 0.01 ^{a,b,c,d}	0.09 ± 0.01 ^{b,c,d}	0.09 ± 0.00 ^{b,c,d}	n.d.	0.06 ± 0.01 ^{a,b}	0.07 ± 0.01 ^{a,b,c}	0.08 ± 0.02 ^{a,b,c,d}	0.07 ± 0.02 ^{a,b,c,d}	n.d.	0.05 ± 0.00 ^{a}	0.05 ± 0.00 ^{a}	0.05 ± 0.00 ^{a}	0.610	0.000	0.540
3,5-Octadien-2-one	MS, KI	n.d.	n.d.	n.d.	n.d.	n.d.	n.d.	0.02 ± 0.00	0.06 ± 0.06	0.02 ± 0.00	0.02 ± 0.01	0.02 ± 0.00	0.02 ± 0.01	0.04 ± 0.03	0.02 ± 0.00	0.02 ± 0.00	n.d.	n.d.	0.02 ± 0.00	n.d.	0.02 ± 0.00	n.d.	n.d.	n.d.	n.d.	0.02 ± 0.01	0.000	0.002	0.000
6,10-Dimethyl-5,9-undecadien-2-one	MS, KI	n.d.	n.d.	n.d.	n.d.	n.d.	0.05 ± 0.02 ^{f}	n.d.	n.d.	n.d.	0.04 ± 0.01 ^{e,f}	0.03 ± 0.01 ^{b,c,d,e}	0.04 ± 0.01 ^{d,e,f}	0.01 ± 0.00 ^{a}	0.02 ± 0.00 ^{a,b,c,d,e}	0.03 ± 0.01 ^{c,d,e}	0.03 ± 0.01 ^{a,b,c,d,e}	0.02 ± 0.00 ^{a,b,c,d}	0.02 ± 0.00 ^{a,b,c}	0.02 ± 0.00 ^{a,b,c}	0.03 ± 0.02 ^{a,b,c,d,e}	0.03 ± 0.01 ^{a,b,c,d,e}	0.02 ± 0.00 ^{a,b,c}	0.01 ± 0.00 ^{a,b,c}	0.01 ± 0.00 ^{a,b}	0.01 ± 0.00 ^{a,b,c}	0.051	0.863	0.225
beta-Ionone	MS, KI	n.d.	n.d.	n.d.	n.d.	n.d.	n.d.	n.d.	n.d.	n.d.	n.d.	0.03 ± 0.01 ^{b,c}	0.03 ± 0.01 ^{c}	n.d.	0.02 ± 0.00 ^{a,b}	0.03 ± 0.01 ^{b,c}	n.d.	0.03 ± 0.00 ^{a,b,c}	n.d.	0.02 ± 0.00 ^{a}	0.03 ± 0.01 ^{c}	n.d.	0.02 ± 0.00 ^{a}	0.02 ± 0.00 ^{a}	0.02 ± 0.00 ^{a}	n.d.	0.003	0.001	0.002
Hydrocarbons																													
Octane	MS, KI	n.d.	n.d.	n.d.	n.d.	n.d.	n.d.	0.02 ± 0.00 ^{a,b}	0.02 ± 0.00 ^{a,b}	n.d.	0.05 ± 0.00 ^{d}	0.02 ± 0.01 ^{b}	0.04 ± 0.00 ^{c}	0.01 ± 0.00 ^{a}	n.d.	0.04 ± 0.01 ^{c}	0.02 ± 0.01 ^{b}	n.d.	n.d.	n.d.	0.04 ± 0.00 ^{c}	n.d.	n.d.	n.d.	n.d.	n.d.	0.001	0.000	0.000
Styrene	MS, KI	n.d.	n.d.	n.d.	n.d.	n.d.	n.d.	n.d.	n.d.	n.d.	n.d.	n.d.	n.d.	n.d.	n.d.	n.d.	n.d.	n.d.	n.d.	n.d.	n.d.	n.d.	0.02 ± 0.00	0.03 ± 0.01	0.01 ± 0.00	0.01 ± 0.00	-	-	-
Esters																													
Ethyl Acetate	MS	n.d.	n.d.	n.d.	n.d.	n.d.	n.d.	n.d.	n.d.	n.d.	n.d.	n.d.	n.d.	n.d.	n.d.	n.d.	n.d.	n.d.	n.d.	n.d.	n.d.	0.06 ± 0.01	0.03 ± 0.00	0.02 ± 0.00	0.03 ± 0.01	0.02 ± 0.01	-	-	-
trans-2-Heptenyl acetate	MS	n.d.	n.d.	n.d.	n.d.	n.d.	n.d.	n.d.	n.d.	n.d.	n.d.	n.d.	n.d.	n.d.	n.d.	n.d.	n.d.	0.06 ± 0.04 ^{a,b}	n.d.	0.08 ± 0.02 ^{b}	n.d.	n.d.	n.d.	n.d.	0.05 ± 0.01 ^{a}	n.d.	0.013	0.005	0.005
Methyl salicylate	MS	n.d.	n.d.	n.d.	n.d.	n.d.	n.d.	n.d.	n.d.	n.d.	n.d.	0.03 ± 0.00 ^{c,d}	0.03 ± 0.01 ^{b,c,d}	0.01 ± 0.00 ^{a}	0.03 ± 0.00 ^{a,b,c}	0.03 ± 0.00 ^{b,c}	0.05 ± 0.01 ^{e}	0.06 ± 0.01 ^{e}	0.03 ± 0.00 ^{b,c,d}	0.02 ± 0.00 ^{a,b}	0.03 ± 0.00 ^{a,b,c}	0.04 ± 0.01 ^{d}	n.d.	0.03 ± 0.00 ^{a,b,c}	0.03 ± 0.00 ^{a,b,c}	0.03 ± 0.00 ^{b,c,d}	0.001	0.002	0.011
Acetic acid octyl ester	MS, KI	n.	n.	n.	n.	n.d.	n.d.	n.d.	n.d.	n.d.	n.d.	n.d.	n.d.	n.d.	n.d.	n.d.	n.d.	0.05 ± 0.03 ^{b}	n.d.	n.d.	n.d.	0.04 ±	0.04 ± 0.01 ^{b}	0.03 ±	0.03 ±	0.02 ±	0.0	0.008	0.008

		d.	d.	d.	d.																	0.01 ^{a,b}		0.01 ^{a,b}	0.01 ^{a,b}	0.01 ^{a}	19		
Terpenes																													
Carvone	MS, KI	n.d.	n.d.	n.d.	n.d.	n.d.	0.08 ± 0.11 ^{b}	0.02 ± 0.00 ^{a,b}	n.d.	n.d.	n.d.	0.02 ± 0.00 ^{a,b}	0.02 ± 0.00 ^{a,b}	0.01 ± 0.00 ^{a}	0.02 ± 0.00 ^{a,b}	0.01 ± 0.00 ^{a,b}	n.d.	n.d.	0.01 ± 0.00 ^{a,b}	0.01 ± 0.00 ^{a,b}	0.01 ± 0.00 ^{a,b}	n.d.	n.d.	n.d.	n.d.	n.d.	0.000	0.000	0.000
.alpha.-Farnesene	MS, KI	n.d.	n.d.	n.d.	n.d.	n.d.	n.d.	n.d.	n.d.	n.d.	n.d.	n.d.	n.d.	n.d.	n.d.	n.d.	n.d.	n.d.	n.d.	n.d.	n.d.	n.d.	n.d.	0.01 ± 0.00	0.01 ± 0.00	0.01 ± 0.00	-	-	-
Sulfur compounds																													
Diallyl sulfide	MS	0.09 ± 0.00	0.09 ± 0.00	0.09 ± 0.00	0.09 ± 0.00	0.09 ± 0.00	0.36 ± 0.47	0.09 ± 0.00	0.34 ± 0.44	0.35 ± 0.47	0.09 ± 0.00	0.08 ± 0.00	0.08 ± 0.00	0.07 ± 0.00	0.08 ± 0.00	0.07 ± 0.00	0.07 ± 0.00	0.07 ± 0.01	0.07 ± 0.00	0.08 ± 0.03	0.07 ± 0.01	0.06 ± 0.01	0.06 ± 0.00	0.06 ± 0.00	0.05 ± 0.00	0.06 ± 0.00	0.008	0.067	0.777
Diallyl disulfide	MS	0.48 ± 0.03	0.48 ± 0.03	0.48 ± 0.03	0.48 ± 0.03	0.48 ± 0.03	n.d.	n.d.	0.55 ± 0.03	n.d.	0.51 ± 0.02	n.d.	n.d.	n.d.	n.d.	n.d.	n.d.	n.d.	n.d.	n.d.	n.d.	n.d.	n.d.	n.d.	n.d.	n.d.	-	-	-
Dipropyl disulfide	MS	0.07 ± 0.00 ^{a,b,c,d,e}	0.07 ± 0.00 ^{a,b,c,d,e}	0.07 ± 0.00 ^{a,b,c,d,e}	0.07 ± 0.00 ^{a,b,c,d,e}	0.07 ± 0.00 ^{a,b,c,d,e}	0.07 ± 0.01 ^{b,c,d,e}	0.08 ± 0.01 ^{d,e}	0.08 ± 0.01 ^{e}	0.07 ± 0.00 ^{a,b,c,d,e}	0.08 ± 0.01 ^{e}	0.08 ± 0.01 ^{e}	0.08 ± 0.02 ^{c,d,e}	n.d.	0.06 ± 0.00 ^{a,b,c,d}	0.07 ± 0.00 ^{a,b,c,d,e}	0.05 ± 0.01 ^{a}	0.06 ± 0.00 ^{a,b,c,d}	0.06 ± 0.00 ^{a,b,c}	0.05 ± 0.01 ^{a,b}	0.06 ± 0.00 ^{a,b,c,d}	0.05 ± 0.00 ^{a}	0.05 ± 0.01 ^{a}	0.05 ± 0.00 ^{a,b}	0.05 ± 0.01 ^{a}	0.06 ± 0.00 ^{a,b}	0.001	0.685	0.066
Other compounds																													
1-Methylpyrrole	MS, KI	n.d.	n.d.	n.d.	n.d.	n.d.	n.d.	n.d.	n.d.	n.d.	n.d.	n.d.	n.d.	n.d.	n.d.	n.d.	n.d.	n.d.	n.d.	n.d.	n.d.	n.d.	n.d.	n.d.	0.01 ± 0.00	0.02 ± 0.00	-	-	-
Phenol	MS	0.13 ±	0.13 ±	0.13 ±	0.13 ±	0.13 ± 0.02 ^{a,b,c}	0.24 ± 0.07 ^{d,e}	0.25 ± 0.04 ^{e}	0.21 ± 0.04 ^{c,d,e}	0.24 ± 0.06 ^{e}	0.18 ± 0.04 ^{a,b,c,d,e}	0.20 ± 0.03 ^{b,c,d,e}	0.26 ± 0.05 ^{e}	0.11 ± 0.02 ^{a}	0.13 ± 0.02 ^{a,b,c}	0.12 ± 0.02 ^{a,b}	0.18 ± 0.01 ^{a,b,c,d,e}	0.19 ± 0.04 ^{a,b,c,d,e}	n.d.	n.d.	0.15 ± 0.01 ^{a,b,c,d}	n.d.	n.d.	n.d.	n.d.	n.d.	0.250	0.001	0.084

		0.02 ^{a,b,c}	0.02 ^{a,b,c}	0.02 ^{a,b,c}	0.02 ^{a,b,c}																								
2-Penthylfuran	MS	2.23 ± 0.13 ^{c}	2.23 ± 0.13 ^{c}	2.23 ± 0.13 ^{c}	2.23 ± 0.13 ^{c}	2.23 ± 0.13 ^{c}	2.11 ± 0.67 ^{c}	0.08 ± 0.01 ^{a}	2.03 ± 0.79 ^{c}	2.55 ± 0.08 ^{c}	0.08 ± 0.01 ^{a}	n.d.	n.d.	1.02 ± 0.40 ^{b}	0.08 ± 0.00 ^{a}	0.06 ± 0.00 ^{a}	n.d.	n.d.	n.d.	n.d.	n.d.	n.d.	n.d.	n.d.	n.d.	n.d.	0.000	0.000	0.000
2-Methylphenol	MS, KI	0.05 ± 0.01 ^{b,c,d}	0.05 ± 0.01 ^{b,c,d}	0.05 ± 0.01 ^{b,c,d}	0.05 ± 0.01 ^{b,c,d}	0.05 ± 0.01 ^{b,c,d}	0.07 ± 0.01 ^{d,e}	0.07 ± 0.01 ^{d,e}	0.07 ± 0.00 ^{d,e}	0.07 ± 0.00 ^{d,e}	0.07 ± 0.01 ^{c,d,e}	0.07 ± 0.00 ^{d,e}	0.08 ± 0.00 ^{e}	n.d.	0.06 ± 0.01 ^{c,d,e}	0.06 ± 0.00 ^{c,d,e}	0.07 ± 0.01 ^{d,e}	0.07 ± 0.01 ^{d,e}	n.d.	0.05 ± 0.01 ^{b,c}	0.05 ± 0.00 ^{c,d}	0.06 ± 0.02 ^{c,d,e}	n.d.	0.02 ± 0.00 ^{a}	n.d.	0.03 ± 0.01 ^{a,b}	0.069	0.000	0.322
4-Methylphenol	MS, KI	0.09 ± 0.01	0.09 ± 0.01	0.09 ± 0.01	0.09 ± 0.01	0.09 ± 0.01	0.14 ± 0.03	0.12 ± 0.03	0.53 ± 0.70	n.d.	0.12 ± 0.02	0.12 ± 0.01	0.13 ± 0.01	0.26 ± 0.35	0.09 ± 0.02	0.12 ± 0.02	0.12 ± 0.02	0.11 ± 0.01	n.d.	0.09 ± 0.01	0.12 ± 0.03	0.10 ± 0.03	n.d.	n.d.	0.09 ± 0.04	0.07 ± 0.02	0.616	0.941	0.727
2-Methoxyphenol	MS	n.d.	n.d.	n.d.	n.d.	n.d.	n.d.	n.d.	n.d.	n.d.	n.d.	n.d.	0.53 ± 0.02 ^{b,c}	n.d.	0.49 ± 0.04 ^{a,b,c}	0.51 ± 0.02 ^{b,c}	n.d.	0.58 ± 0.04 ^{c}	n.d.	0.52 ± 0.03 ^{b,c}	0.51 ± 0.01 ^{b,c}	0.42 ± 0.04 ^{a}	n.d.	0.54 ± 0.01 ^{b,c}	0.47 ± 0.12 ^{a,b}	n.d.	0.113	0.000	0.139
3,5-Dimethylphenol	MS	n.d.	n.d.	n.d.	n.d.	n.d.	n.d.	n.d.	n.d.	n.d.	n.d.	0.03 ± 0.01 ^{a,b}	0.02 ± 0.00 ^{a,b}	n.d.	0.03 ± 0.00 ^{a,b}	0.03 ± 0.01 ^{a,b}	0.05 ± 0.01 ^{c}	n.d.	n.d.	n.d.	0.04 ± 0.02 ^{b,c}	n.d.	0.01 ± 0.00 ^{a}	n.d.	n.d.	n.d.	0.637	0.004	0.035
2-Methoxy-4-methylphenol	MS	n.d.	n.d.	n.d.	n.d.	n.d.	0.08 ± 0.01 ^{b,c}	0.07 ± 0.02 ^{a,b,c}	0.07 ± 0.00 ^{a,b,c}	0.07 ± 0.01 ^{a,b,c}	0.06 ± 0.01 ^{a,b,c}	0.07 ± 0.01 ^{a,b,c}	0.08 ± 0.00 ^{b,c}	0.07 ± 0.01 ^{a,b,c}	0.05 ± 0.01 ^{a,b}	0.07 ± 0.01 ^{a,b,c}	0.06 ± 0.01 ^{a,b,c}	0.08 ± 0.02 ^{b,c}	0.06 ± 0.01 ^{a,b,c}	0.07 ± 0.01 ^{a,b,c}	0.06 ± 0.01 ^{a,b,c}	0.08 ± 0.01 ^{c}	0.05 ± 0.00 ^{a,b,c}	0.06 ± 0.00 ^{a,b,c}	0.06 ± 0.00 ^{a,b,c}	0.05 ± 0.00 ^{a}	0.355	0.114	0.837
4-Ethyl-2-methoxyphenol	MS, KI	0.06 ± 0.	0.06 ± 0.	0.06 ± 0.	0.06 ± 0.	0.06 ± 0.01 ^{a,b}	0.08 ± 0.03 ^{a,b}	0.08 ± 0.04 ^{a,b}	0.08 ± 0.01 ^{a,b}	0.08 ± 0.00 ^{a,b}	0.07 ± 0.02 ^{a,b}	0.07 ± 0.01 ^{a,b}	0.08 ± 0.01 ^{a,b}	0.07 ± 0.01 ^{a,b}	0.05 ± 0.00 ^{a,b}	0.09 ± 0.02 ^{b}	0.07 ± 0.01 ^{a,b}	0.07 ± 0.01 ^{a,b}	0.06 ± 0.01 ^{a,b}	0.06 ± 0.00 ^{a,b}	0.08 ± 0.02 ^{a,b}	0.07 ± 0.02 ^{a,b}	n.d.	0.05 ± 0.00 ^{a,b}	0.05 ± 0.00 ^{a,b}	0.04 ± 0.01 ^{a}	0.244	0.393	0.955

		01^{a,b}	01^{a,b}	01^{a,b}	01^{a,b}																								
2,6-Dimethoxyphenol	MS	0.10 ± 0.02	0.10 ± 0.02	0.10 ± 0.02	0.10 ± 0.02	0.10 ± 0.02	0.21 ± 0.15	0.26 ± 0.24	0.12 ± 0.02	0.11 ± 0.02	0.17 ± 0.09	0.13 ± 0.05	0.17 ± 0.05	0.08 ± 0.01	0.07 ± 0.01	0.17 ± 0.07	0.12 ± 0.03	0.12 ± 0.05	0.05 ± 0.03	0.06 ± 0.01	0.21 ± 0.14	0.13 ± 0.11	n.d.	n.d.	n.d.	0.04 ± 0.01	0.809	0.254	0.562
2-Methoxy-4-(2-propenyl)-	MS, KI	n.d.	n.d.	n.d.	n.d.	n.d.	n.d.	n.d.	n.d.	n.d.	n.d.	0.01 ± 0.00	0.02 ± 0.01	n.d.	n.d.	0.02 ± 0.01	0.02 ± 0.00	0.02 ± 0.01	n.d.	n.d.	0.03 ± 0.02	n.d.	n.d.	n.d.	n.d.	n.d.	0.433	0.391	0.509

Data is displayed as mean ± standard deviation (x10^6). In the statistical model, the fermentation time (TIME) and the microbial treatment (TREATMENT) were considered as fixed terms and the effect of both along with the interaction (TIME × TREATMENT) were assessed using a two-WAY Analysis of Variance (ANOVA). Statistical differences ($p \leq 0.05$) among the different samples are marked with letters as superscripts ($^{a-i}$). Not detected (n.d.). Identification method (Id.): Mass spectrometry (MS); Kovats Index (KI). Experimental groups 1 (Control), 2 (inoculated with lactic acid bacteria 116), 3 (inoculated with LAB 205), 4 (coinoculated with LAB 116+205), and 5 (inoculated with the commercial culture).
